# Gastric epithelial neoplasm of fundic-gland mucosa lineage: representative of the low atypia differentiated gastric tumor and Ki67 may help in their identification

**DOI:** 10.3389/pore.2024.1611734

**Published:** 2024-05-30

**Authors:** Houqiang Li, Lanqing Zheng, Guodong Zhong, Xunbin Yu, Xia Zhang, Linying Chen, Xin Chen

**Affiliations:** ^1^ Shengli Clinical Medical College of Fujian Medical University, Fuzhou, China; ^2^ Fujian Provincial Hospital, Fuzhou, China; ^3^ The Second Affiliated Hospital of Fujian Traditional Chinese Medical University, Fuzhou, China; ^4^ The First Affiliated Hospital of Fujian Medical University, Fuzhou, Fujian, China

**Keywords:** gastric cancer, gastric adenocarcinoma of fundic-gland type, gastric adenocarcinoma of fundic-gland mucosa type, oxyntic gland adenoma, low-grade differentiated gastric tumors

## Abstract

**Background:**

Gastric epithelial neoplasm of the fundic-gland mucosa lineages (GEN-FGMLs) are rare forms of gastric tumors that encompass oxyntic gland adenoma (OGA), gastric adenocarcinoma of the fundic-gland type (GA-FG), and gastric adenocarcinoma of the fundic-gland mucosa type (GA-FGM). There is no consensus on the cause, classification, and clinicopathological features of GEN-FGMLs, and misdiagnosis is common because of similarities in symptoms.

**Methods:**

37 cases diagnosed with GEN-FGMLs were included in this study. H&E-stained slides were reviewed and clinicopathological parameters were recorded. Immunohistochemical staining was conducted for MUC2, MUC5AC, MUC6, CD10, CD56, synaptophysin, chromograninA, p53, Ki67, pepsinogen-I, H^+^/K^+^-ATPase and Desmin.

**Results:**

The patients’ ages ranged from 42 to 79 years, with a median age of 60. 17 were male and 20 were female. Morphologically, 19 OGAs, 16 GA-FGs, and two GA-FGMs were identified. Histopathological similarities exist between OGA, GA-FG, and GA-FGM. The tumors demonstrated well-formed glands, expanding with dense growth patterns comprising pale, blue-grey columnar cells with mild nuclear atypia. These cells resembled fundic gland cells. None of the OGA invaded the submucosal layer. The normal gastric pit epithelium covered the entire surface of the OGA and GA-FG, but the dysplasia pit epithelium covered the GA-FGM. Non-atrophic gastritis was observed in more than half of the background mucosa. All cases were diffusely positive for MUC6 and pepsinogen-I on immunohistochemistry. H^+^/K^+^-ATPase staining was negative or showed a scattered pattern in most cases. MUC5AC was expressed on the surface of GA-FGMs. p53 was focally expressed and the Ki67 index was low (1%–20%). Compared with OGA, GA-FG and GA-FGM were more prominent in the macroscopic view (*p* < 0.05) and had larger sizes (*p* < 0.0001). Additionally, GA-FG and GA-FGM exhibited higher Ki67 indices than OGA (*p* < 0.0001). Specimens with Ki-67 proliferation indices >2.5% and size >4.5 mm are more likely to be diagnosed with GA-FG and GA-FGM than OGA.

**Conclusion:**

GEN-FGMLs are group of well-differentiated gastric tumors with favourable biological behaviours, low cellular atypia, and low proliferation. Immunohistochemistry is critical for confirming diagnosis. Compared with OGA, GA-FG and GA-FGM have larger sizes and higher Ki67 proliferation indices, indicating that they play a critical role in the identification of GEN-FGML. Pathologists and endoscopists should be cautious to prevent misdiagnosis and overtreatment, especially in biopsy specimens.

## Introduction

Gastric cancer (GC) is one of the most common malignant tumors of the digestive system worldwide [1]. Benefiting from the improvement and popularization of endoscopic technology in recent years, researchers have discovered many low-grade well differentiated gastric tumors, such as the gastric epithelial neoplasm of the fundic-gland mucosa lineages (GEN-FGML). GEN-FGMLs are rare tumors that differentiate from gastric mucosa to fundic glands, including oxyntic gland adenoma (OGA), gastric adenocarcinoma of the fundic-gland type (GA-FG), and gastric adenocarcinoma of the fundic-gland mucosa type (GA-FGM) [2–4]. OGA and GA-FG are defined as well-differentiated neoplasms composed of parietal cell-like tumor cells with mild morphologic atypia that are positive for pepsinogen I and/or H^+^/K^+^-ATPase. GA-FG can be divided into three subcategories according to the tumor composition: chief cell predominant, parietal cell predominant, and mixed phenotype [2]. The histologic features of GA-FGM are similar to those of GA-FG, but the gastric pit epithelium is atypical and malignant [5, 6]. GA-FGM was also divided into three subtypes (Type 1, Type 2, and Type 3) according to the mucosal structure of the pit epithelium and fundic gland [4]. GEN-FGML can be easily misdiagnosed. GA-FG is known as an oxyntic mucosal polyp/adenoma in the West [7], whereas Japanese scholars believe that OGA is the intramucosal stage of GA-FG. Japanese scholars divided fundic gland tumors into GA-FG and GA-FGM, and GA-FGM is believed to have more malignant potential [6, 8]. In addition, OGA and GA-FG were officially listed as new gastric neoplasms in the World Health Organization (WHO) classification of tumors in 2019 [9]. GA-FG is diagnosed when OGA invades the submucosal layer. GEN-FGMLs, particularly OGA and GA-FG, have a good prognosis [2]. Unlike other low-grade well differentiated gastric tumors, GEN-FGMLs have a high frequency of GNAS mutations, which are a characteristic genetic feature of GEN-FGMLs [4]. Here, we report the clinicopathological features of 37 cases of GEN-FGMLs, helping pathologists and endoscopists diagnose GEN-FGMLs.

## Materials and methods

### Data collection

Between January 2019 and December 2023, 43 specimens from 37 patients pathologically diagnosed with GEN-FGMLs, including 19 OGAs, 16 GA-FGs, and two GA-FGMs, were included from Fujian Provincial Hospital. All original H&E slides were reviewed, and patients’ clinicopathologic parameters were extracted from the hospital medical record system. These parameters included age, sex, tumor location, morphology, depth of invasion, vascular invasion, and nerve invasion. The background mucosa was also recorded. The diagnosis of the disease were based on the 5th edition of the WHO Health Organization tumor classification and the 6th edition of the Japanese Gastric Carcinoma Classification. All pathologic diagnoses were reviewed by 2 senior pathologists and were followed up until April 2024.

### Histology and immunohistochemistry

Histopathological analyses were performed on the basis of the following factors: histological subcategories (OGA, GA-FG, and GA-FGM), architectural patterns, presence of cytonuclear atypia, depth of invasion, and atrophy or intestinal metaplasia of the background. The presence of MUC2, CD10, MUC5AC, MUC6, Ki67, chromograninA (CgA), synaptophysin (Syn), CD56, p53, pepsinogen-I, H^+^/K^+^-ATPase, and Desmin was measured using specific antibodies and analyzed using the Lumatas platform (Maixin Biotechnology Co. LTD, China). Immunostaining results were considered positive if ≥ 10% of neoplastic cells were stained. Details of the antibodies, incubation conditions, and antigen retrieval are listed in Supplementary Table S1. PBS was used as a negative control. The histopathological diagnosis and immunostaining results were confirmed by two pathologists. A consensus review under a multi-head microscope was performed when facing inconsistent results.

### Statistical analysis

Graphs and statistical analysis were administered using GraphPad Prism 9.0 (La Jolla, CA, United States). Continuous data were compared using the Mann-Whitney U test. Categorical variables were analyzed using Fisher’s exact test. A *p*-value < 0.05 was considered statistically significant.

## Results

### Clinicopathologic characterization of GEN-FGMLs

**FIGURE 1:**
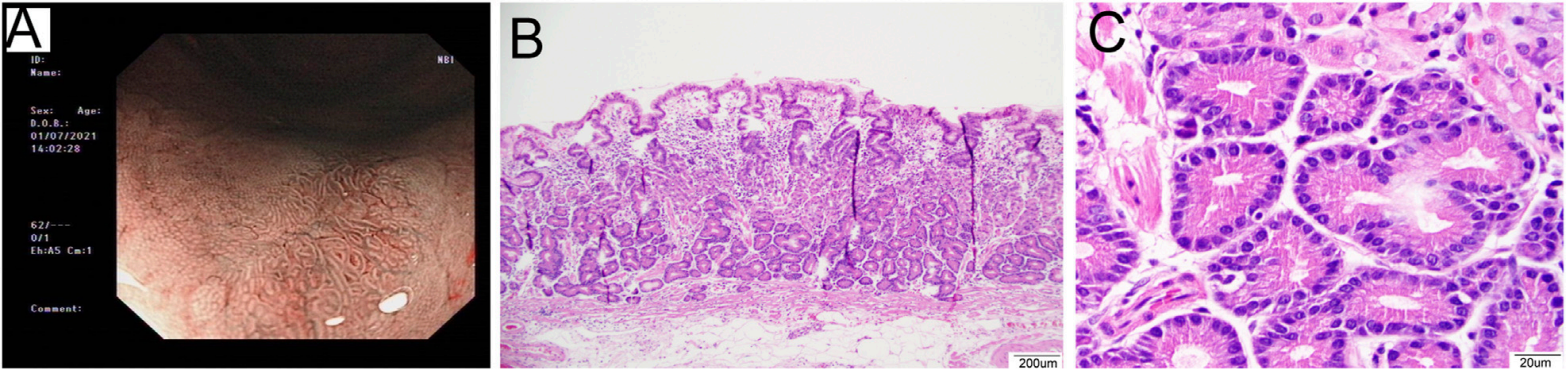
Representative images of a 53-year-old male patient diagnosed with OGA. **(A)**: The image of NBI shows fundus mucosa observed as a flat lesion in the macroscopic view. **(B)**: The lesion was clearly defined on the mucosa layer. The lesion covered normal gastric pit epithelium and glands exhibited branching, expanding arrangement (40×). **(C)**: The glands were composed of columnar cells with mild nuclear atypia, similar to fundic gland cells. The nuclei were slightly enlarged (400×).

**FIGURE 2:**
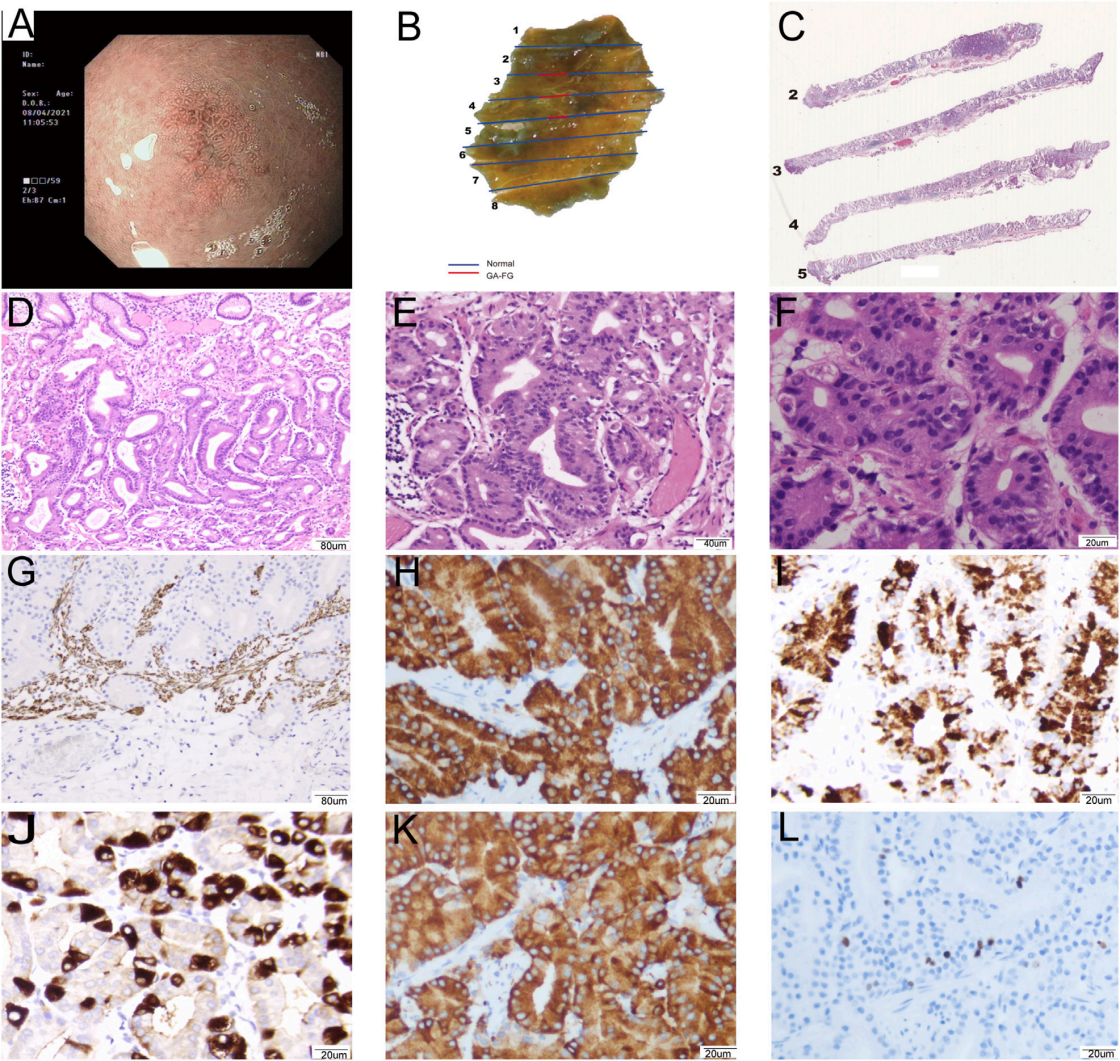
Representative images of a 63-year-old female patient diagnosed with GA-FG: 0-IIb, Tub1>tub2 (SM1), Ly0, V0, UL (−), pHM0, pVM0. **(A)**: The image of NBI shows background fundus mucosa without *H. pylori* infection and non-atrophic gastritis. The 0–IIb lesion had a blurred border with irregularly arranged marginal crypt epithelium with asymmetric distribution. The lesion consisted of microvessels with dendritic vasculature. **(B)**: Macroscopic findings of the resected specimen showing a protruding submucosal lesion. **(C)**: The lesion was clearly defined and infiltrated into the submucosa. **(D)** The lesion covered normal gastric pit epithelium and glands and exhibited branching, expanding arrangement, and fusion (100×). **(E)**: The tumors exhibited mild cellular and structural atypia with well-formed glands, similar to fundic gland cells (200×). **(F)**: The glands were composed of pale grey-blue, basophilic columnar cells with mild nuclear atypia. The nuclei had hyperchromatic chromatin and were slightly enlarged (400×). **(G–L)**: Immunohistochemical analysis. **(G)** Desmin showed carcinoma invasion of the submucosal layer (100×). Carcinoma was diffusely positivity for MUC6 (H, 400×), pepsinogen-I (I, 400×), and Syn (K, 400×), focally positivity for H+/K + -ATPase (J, 400×), and Ki67 index was 3% (L, 400×).

**FIGURE 3:**
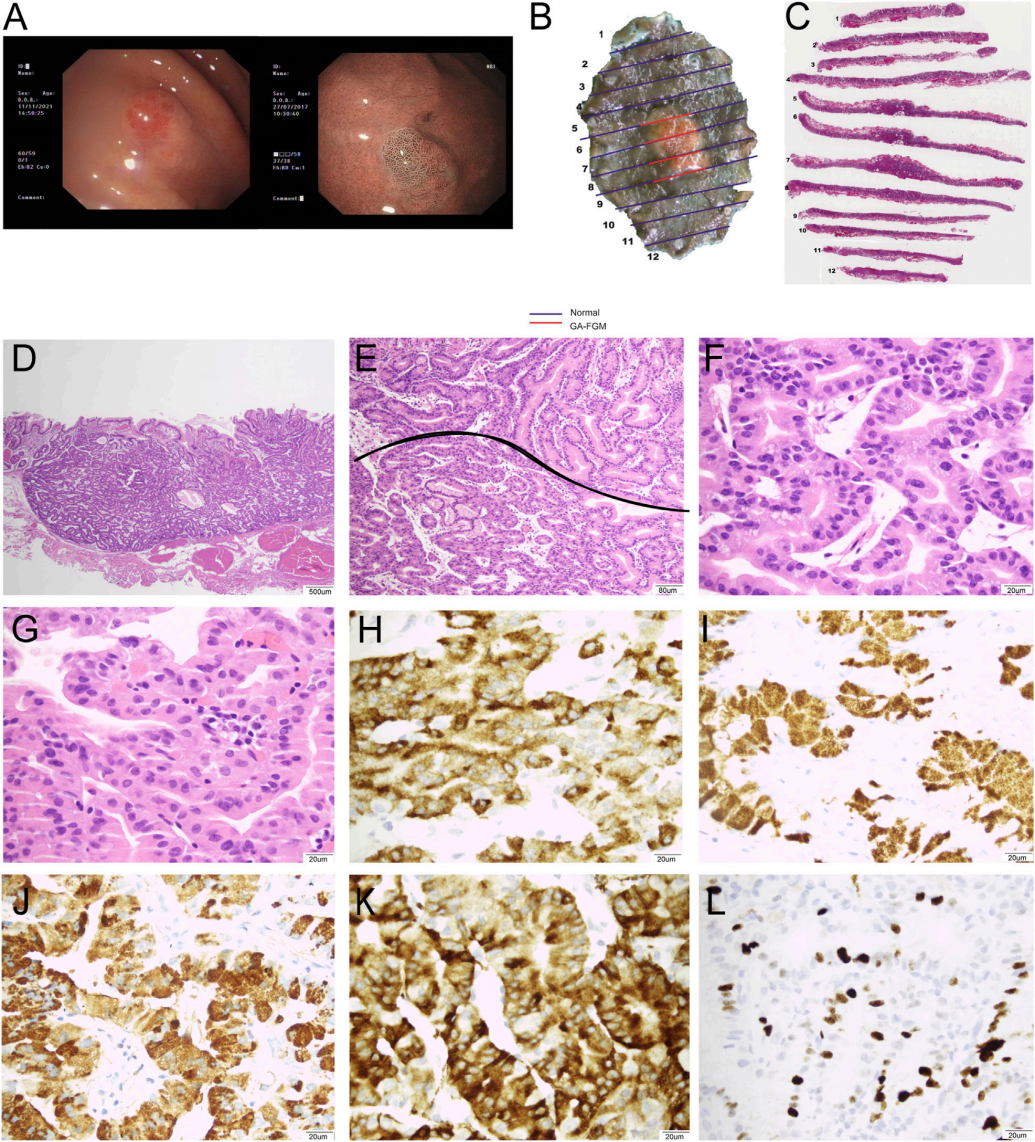
Representative images of a 71-year-old female patient diagnosed with GA-FGM: 0-IIa, Tub1>tub2 (SM2), Ly0, V0, UL (−), pHM0, and pVM0. **(A)**: Conventional endoscopy revealed a smooth, reddish lesion in the gastric fundus with distinct borders. The background mucosa did not show *H. pylori* infection and non-atrophic gastritis. The lesion exhibited significant changes in microvascular and microsurface patterns. The marginal crypt epithelium had a complex ripple-like pattern that was wider and irregularly arranged compared to that of the background mucosa. **(B,C)**: Macroscopic findings of the resected specimen showing a protruding lesion. **(C,D)**: The lesion was well-defined and infiltrated into the submucosa (20×). **(E)**: The pit epithelium exhibited cellular atypia, and glands exhibited branching, expanding arrangement, and fusion (400×). **(F)**: The tumors exhibited marked cellular and structural atypia. The glands were composed of pale grey-blue and basophilic columnar cells (40×). **(G)**: The pit epithelium exhibited obvious cellular and structural atypia (400×). **(H–L)**: Immunohistochemical analysis (400×). Carcinoma was diffusely positive for MUC6 **(H)** and pepsinogen-I **(I)**. The pit epithelium was positive for MUCAC **(J)** and diffusely positive for Syn **(K)**, and Ki67 index was 15% **(L)**.

All patients were 42–79 years old, with a median age of 60. Of all patients, 17 were male (45.95%) and 20 were female (54.05%), and the male-to-female ratio was approximately 4: 5. Endoscopic findings indicated 15 lesions in the fundus and 22 in the gastric body. 12 cases of OGA and five cases of GA-FG were observed as flatting lesions in the macroscopic view (Figure 1A). While, six cases of OGA, 11 cases of GA-FG, and all two cases of GA-FGM were observed as protruding lesions in the macroscopic view (Figures 2A, B; 3A, B). Of all GEN-FGMLs, 36 cases were available for the follow-up survey, 15 cases were biopsied, 22 cases were endoscopically resected, and one case underwent additional surgical resection. Endoscopic resection was performed in 17 patients with GA-FG and GA-FGM. The average follow-up time for cases in this group was 20.6 months (ranging from 5 to 41 months). During the follow-up period, one patient of GA-FG presented with 1 lymph node metastasis and underwent additional surgical resection. There was no recurrence, metastasis, or gastric cancer-specific death in all others (Table 1).

**TABLE 1:** Clinicopathological characteristics, immunohistochemical analysis of GA-FGMLs.

Clinicopathological characteristics	OGA (*n* = 19)	GA-FG/GA-FGM (*n* = 18)	*p-val*
Sex (male: female)	8:11	9:9	0.75
Age (Median: years)	61 (range: 42–69)	58 (range: 45–79)	0.49
Location	B: F = 13:6	B: F = 9:9	0.32
Macroscopic type (Flat: Protruded)	12:6	5:13	0.044
Size of tumor (average: mm)	3.8 (2–8), 18cases	8.7 (4–17), 17cases	<0.0001
Depth of invasion (SM1:SM2)	NA	16:2	NA
Lymphatic and venous invasion	NA	5.88% (1/17)	NA
Perineural invasion	NA	0% (0/17)	NA
Atrophic gastritis	43.75% (7/16)	44.44% (8/18)	>0.99
Follow up time (months)	20.7 (range: 5–41)	20.5 (range: 5–39)	>0.99
Outcome	19 cases: SWD	17 cases: SWD Lymph node metastasis in one case	NA
Immunohistochemical analysis			
pepsinogen-I	100% (11/11)	100% (14/14)	>0.99
H/K-ATPase	0% (0/11)	14.3% (2/14)	0.50
MUC2	0% (0/11)	0% (0/16)	>0.99
MUC5AC	0% (0/11)	12.5% (2/16)	0.51
MUC6	100% (11/11)	100% (16/16)	>0.99
CD10	0% (0/10)	0% (0/16)	>0.99
CgA	0% (0/11)	0% (0/15)	>0.99
Syn	100% (11/11)	100% (15/15)	>0.99
CD56	100% (11/11)	100% (15/15)	>0.99
p53	4.7% (1%–20%), 14 cases	4.2% (1%–30%), 15 cases	>0.99
Ki-67	1.9% (1%–5%), 16 cases	7.5% (2%–20%), 16 cases	<0.0001

B: gastric body; F: fundus; NA: date not available; SWD: survive without disease.

Of all patients, 14 were diagnosed with OGA due to limitations in evaluating mucosal layer depth, 16 were diagnosed with GA-FG, and two cases were diagnosed with GA-FGM. Four patients were misdiagnosed with OGA based on pathological biopsy before endoscopic resection. The tumors classified as GEN-FGMLs were solitary, with sizes ranging from 2 mm to 17 mm (average = 6.3 mm). The average size of GA-FG and GA-FGM tumours is larger than OGA tumours (3.8 mm for OGA vs. 8.7 mm for GA-FG and GA-FGM, *p* < 0.0001). Moreover, OGA had a flattened form, whereas GA-FG and GA-FGM commonly had a protruded form (*p* = 0.044). No significant differences were found between OGA, GA-FG, and GA-FGM with respect to other variables, such as sex, age, location, and atrophic gastritis of the peripheral mucosa (Table 1).

**FIGURE 4:**
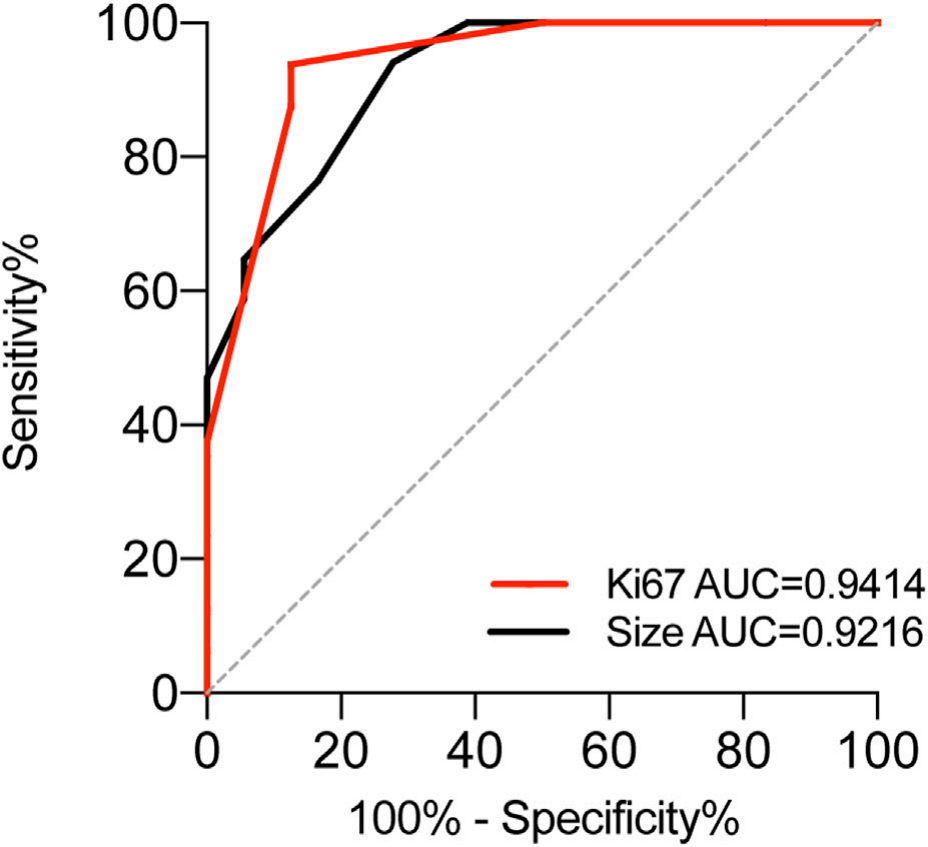
ROC analysis to assess the specificity and sensitivity of Ki-67 and Size to differentiate between OGA GA-FG and GA-FGM in GA-FGML.

From a histological perspective, OGA, GA-FG, and GA-FGM have similar morphologies. At low magnification, all of the tumors showed an expansive and dense growth pattern, with clear demarcation, but without migrating (Figures 1B, 2C, 3C). Normal gastric pit epithelium covered the entire surface of OGA and GA-FG (Figures 1B, 2D). However, dysplastic/cancerous pit epithelium covered GA-FGM (Figure 3E). At high magnification, GEN-FGMLs tumor cell exhibited mild cellular and structural atypia with well-formed glands displaying expansive and dense growth patterns. The glands were composed of pale gray-blue basophilic columnar cells with mild nuclear atypia, similar to fundic gland cells. The glands exhibited branching, expansion, a back-to-back arrangement, and fusion. The nuclei were arranged in a crowded round or ovoid shape, with small nucleoli, hyperchromatic chromatin, and slight enlargement (Figures 1C, 2E, F). However, GA-FGM showed more atypia than GA-FG and OGA (Figures 3F, G). Mitosis and necrosis were not observed in any case. IHC staining showed that MUC6 and pepsinogen I were positive in all cases (Figures 2H, I). While the H^+^/K^+^-ATPase staining was negative or showed a scattered pattern (<10% of neoplastic cells were stained) in most of the cases. Positive staining was observed in one GA-FG and one GA-FGM case (Figure 2J). MUC5AC staining was positive in the pit epithelium and GA-FGM surface tumor cells (Figure 3J). MUC2 and CD10 were negative in all the cases. Regarding neuroendocrine markers, CD56 and Syn were positive in staining (Figures 2K, 3K), but CgA was negative. Additionally, GA-FG and GA-FGM exhibited higher Ki-67 proliferation indices (*p* < 0.0001) than OGA (Table 1) (Figures 2L, 3L). However, no significant findings were reported for other markers. None of the patients exhibited p53 overexpression. The desmin staining (Figure 2G) showed the invasion of the carcinoma into the submucosal layer. GA-FG invaded the submucosal layer at a depth of < 500 μm (SM1) in 15 cases, and GA-FGM invaded the submucosal layer at a depth of > 500 μm (SM2) in one case. Furthermore, We used receiver operating characteristics (ROC) analysis to calculate the optimal cut-off value of the Ki-67 proliferation indices (>2.5%) and lesion size (>4.5 mm) in GEN-FGMLs diagnosis and revealed that the Ki-67 proliferation indices and lesion size serve as a good indicator for differentiating GEN-FGMLs (Figure 4) (Table 2).

**TABLE 2:** Summary of clinicopathologic characteristics of GA-FGMLs.

Parameters	OGA	GA-FG	GA-FGM
Size^a^	≤4.5 mm	>4.5 mm	
Macroscopic type	Flat	Protruded	
Submucosal invasion	Absent	Present	
Growth pattern	Demonstrated expansive and dense growth pattern, with clear demarcation.Tumor glands exhibited branching, expanding arrangement and fusion		
Tumor cell differentiation	Dominated by chief cell differentiation or mixed with parietal cells differentiation		Dominated by chief cell differentiation and mixed with gastric pit epithelium differentiation
Tumor heterogeneity	Exhibited mild cellular and structural atypia, similar to fundic gland cells		Exhibited marked cellular and structural atypia. The pit epithelium is atypical and malignant
Gastric pit epithelium	Normal		Atypical proliferation or carcinogenesis
Synaptophysin	Positive	Positive	
Pepsinogen-I	Positive	Positive	
MUC6	Positive	Positive	
MUC5AC	Negative		Positive
Ki67^b^	≤2.5%	>2.5%	

^a^ Sensitivity% = 94.12%,Specificity% = 72.22%, Youden Index = 0.6634.

^b^ Sensitivity% = 93.75%,Specificity% = 87.5%, Youden Index = 0.8125.

## Discussion

Recently, the incidence of early gastric cancer has increased significantly with the application of narrow-band imaging (NBI) magnifying endoscopy [10]. Many *H. Pylori*-negative gastric adenocarcinomas, including GEN-FGML, have been reported [2, 6, 10–17]. In 2021, GEN-FGML was classified into OGA, GA-FG, and GA-FGM, based on cell differentiation and histological features [4]. Furthermore, GA-FG can be divided into three subcategories according to the tumor composition: chief cell predominant, parietal cell predominant, and mixed phenotype [9]. And GA-FGM was also divided into three subtypes (Type 1, Type 2, and Type 3) according to the mucosal structure of the pit epithelium and fundic gland [4].

In our study, we present 37 cases of GEN-FGMLs, including 19 cases of OGA, 16 cases of GA-FG, and two cases of GA-FGM. Previous studies in Japan have shown that GEN-FGMLs are predominantly observed in males [4]. However, our results showed that GEN-FGMLs are more common in females in China. In the present study, GEN-FGMLs were different based on their sizes. GA-FG and GA-FGM had larger diameters, with an average of 8.8 mm, than OGA, with an average diameter of 5 mm. Furthermore, our study demonstrated that GA-FG and GA-FGM had proliferation indices with Ki-67 than OGA, suggesting that Ki-67 plays a crucial role in the diagnosis of GEN-FGML. Previous studies showed that the background mucosa of GEN-FGML shows no atrophic changes and intestinal metaplasia [4, 16]. In the present study, over 40% of patients had slight atrophic gastritis in the adjacent mucosa.

Histopathological assessment is essential for confirming the diagnosis of GEN-FGMLs. According to the WHO classification, the depth of invasion differentiates between OGA and GA-FG [9]. Based on the Japanese diagnostic criteria, despite showing low-grade atypia and invading the submucosal layer, many stomach neoplasms are diagnosed as invasive carcinomas in the absence of an obvious desmoplastic response [2, 18, 19]. Thus, Japanese scholars have proposed that OGA is the intramucosal stage of GA-FG, and OGA and GA-FG represent different developmental stages of the same tumor [8]. The identification of OGA and GA-FG as intramucosal adenomas or low-grade gastric adenocarcinomas relies on whether the pathologist uses the Western or Japanese diagnostic criteria. In this cohort study, our diagnosis followed the WHO criteria, as we believe that overdiagnosis may cause unnecessary overtreatment in patients in China.

OGA and GA-FG are well-differentiated tumors with expansive and dense growth, composed of pale gray-blue basophilic columnar cells with mild nuclear atypia and slightly enlarged fundic gland cells. IHC staining revealed that they were positive for pepsinogen-I. The surface of the lesions was covered with a normal gastric pit epithelium. In addition, the deep part of the tumor was irregularly branched or dendritic. The tumor gland and background mucosa were demarcated without migrating. The histological features of GA-FGM were similar to those of GA-FG. However, GA-FGM is always accompanied by differentiation to the fundic glands and pit mucous cells and occasionally progresses into tumors consisting of pyloric glands. Differentiation into fundic glands and pit mucous cells suggests that GA-FGM presumably possesses multilineage differentiation potential. GA-FGM is a unique type of complex gastric adenocarcinoma with differentiated fundic glands [2]. Our study revealed histological characteristics similar to those of GEN-FGMLs. Compared to OGA and GA-FG, GA-FGM exhibits greater cellular atypia and malignant transformation of the pit epithelium [6]. GA-FGM showed the highest malignant potential due to higher rates of vascular invasion and lymph node metastasis [2]. Owing to the limited sample size, we did not observe lymphatic invasion and vascular invasion in GA-FGM. However, lymph node metastasis was observed in one GA-FG case. The IHC staining results were consistent with those of previous studies. In the gastric mucosa, pit mucous cells express MUC5AC, gland mucous cells express MUC6, chief cells express pepsinogen I, and parietal cells express H^+^/K^+^-ATPase. GEN-FGMLs mainly consist of tumor components with chief cell-like differentiation, whereas gland mucous cells were previously thought to be the precursors of chief cells [20]. All GEN-FGML samples were positive for MUC6 and pepsinogen-I, indicating that the GEN-FGMLs originated from immature chief cells. MUC5AC was expressed in the pit epithelium and GA-FGM surface, whereas MUC2 and CD10 were not detected. GEN-FGMLs also expressed neuroendocrine markers (Syn and CD56) but not CgA. GEN-FGMLs had a low Ki-67/MIB1 proliferation index and lacked p53 protein.

GEN-FGMLs contain *GNAS* mutation, which is thought to be a characteristic genetic feature [4]. Ueyama *et al.* [4] showed that GEN-FGMLs belong to the same genetic lineage, and that GA-FGM may be the final stage of OGA and GA-FG. In addition to *GNAS* mutations, GEN-FGMLs have genetic mutations associated with *Wnt/β-catenin*, *KRAS*, *PIK3CA*, and sonic hedgehog signalling pathways, but not with *TP53* [6, 21–25]. Thus, *Wnt/β-catenin* signalling pathway may play a role in the development and progression of GEN-FGMLs [6], suggesting that targeting the *Wnt/β-catenin* pathway or *GNAS* mutations may be a potential therapeutic strategy for treating GEN-FGMLs. Further studies are needed to fully understand the molecular mechanisms underlying GEN-FGML.

Previous studies on the origin of GEN-FGMLs have not been consistent and have suggested that spasmolytic polypeptide-expressing metaplasia (SPEM) is fundamental to the development of intestinal metaplasia and gastric cancer, and that mature chief cells cannot dedifferentiate into SPEM. However, recent findings suggest that chief cells undergo differentiation into SPEM [26]. Leushacker *et al.* [27] hypothesized that Lgr5^+^ chief cells are the major origin of gastric cancer because Lgr5^+^ cells play an essential role in maintaining homeostatic stem cells. It has been shown that chief cells can dedifferentiate into different types of gastric mucosal epithelium, such as pit mucosal cells and gland cells. These results suggest that GEN-FGMLs may be derived from malignant transformation of the chief cells. Recent studies have indicated that intramucosal gastric carcinoma (IGC), OGA, and SPEM are derived from common ancestral glands. IGC and OGA originate from the KRAS-mutated SPEM [28]. However, the specific mechanism needs to be investigated further.

It is crucial to distinguish GEN-FGMLs from other gastric tumors. MUC6 and pepsinogen-I co-expression are crucial for the diagnosis of GEN-FGMLs, but the use of simple IHC markers may result in misdiagnosis. All participants in this study were positive for CD56 and Syn, and negative for CgA. These results could have led to the misdiagnosis of neuroendocrine tumors. Gastric neuroendocrine tumors are organ-like structures with basophilic tumor cells, accompanied by significant interstitial microvascular hyperplasia and CgA expression compared to GEN-FGMLs, which do not express CgA [29]. Distinguishing between GEN-FGMLs and pyloric gland adenomas (PGAs) is also challenging. Both PGA and GEN-FGMLs develop in the gastric fundus mucosa and express MUC6, pepsinogen-I, and MUC5AC. Nonetheless, PGAs show polypoid proliferation of pyloric-type glands with cuboidal/columnar cells and foamy ground-glass cytoplasm. Kushima *et al.* revealed that the outer tumor cells in PGAs are taller than the inner cells [25]. Differentiated adenocarcinoma with low atypia was related to GA-FG, but it lacked chief or parietal cells and showed an aggressive growth pattern despite the low atypia. Owing to the rarity of GEN-FGMLs and lack of awareness, most of our cases were primarily misdiagnosed as fundic gland polyps (FGPs). Both FGPs and GEN-FGMLs originate from fundic glands and exhibit similar features, such as the presence of hyperplastic glandular structures, cystic dilation, and distortion of glandular architecture. Typically, FGPs are benign lesions with less than 5 mm in diameter, consisting of dilated oxynitic glands, foveolar hypoplasia, and parietal hyperplasia [9]. However, GEN-FGMLs originate from the fundic glands of the stomach and display glandular or tubular structures with a monoclonal shape. FGPs can also exhibit intraepithelial neoplasia and dysplasia [30]. Notably, dysplasia or adenocarcinoma in fundic gland polyps originates from the foveolar epithelium and not from the fundic glands. It is difficult to diagnose GEN-FGMLs based on biopsy specimens because the mucosal muscle may be indiscernible in such samples. Previous studies and our study have shown that GEN-FGML lesions with larger sizes are more often diagnosed as GA-FG or GA-FGM [4, 16]. Furthermore, our study demonstrated that GA-FG and GA-FGM had higher proliferation indices with Ki-67 than OGA, suggesting that Ki-67 plays a crucial role in the diagnosis of GEN-FGML, especially in biopsy specimens. Specimens with Ki-67 proliferation indices >2.5% and lesion size >4.5 mm are more likely to be diagnosed with GA-FG and GA-FGM than OGA. Moreover, endoscopic findings resemble those of early undifferentiated carcinoma and mucosa-associated lymphoid tissue. Endoscopy plays a limited role in the differential diagnosis. NBI is a promising technique for the diagnosis of GA-FGM and GA-FG. There are distinctive differences between GA-FGM and GA-FG in conventional endoscopy and NBI, including indigo carmine dye spraying, demarcation line, peculiar microvascular pattern, irregular microsurface pattern, and wider marginal crypt epithelium [13].

GEN-FGMLs are lower aggressive than other GCs as they possess minor cellular atypia, less vascular invasion and proliferation, and lack *TP53* mutations. GA-FGM, particularly type 2 GA-FGM, displays the highest malignant potential owing to strong vascular invasion, lymph node metastasis, and deep submucosal invasion [4]. Standard indications for endoscopic and surgical treatment of GEN-FGMLs are urgently needed. Two retrospective analyses from Japan have shown that endoscopic resection is currently the most common and appropriate initial treatment for GEN-FGMLs [4, 16]. Iwamuro *et al.* [16] indicated that GA-FG with an invasion depth of > 500 μm will not recur, even if endoscopic resection is the only treatment, as muscle infiltration and vascular invasion are uncommon in GA-FG. Ueyama *et al.* [4] demonstrated that no cases of GEN-FGMLs (including GA-FGM) that underwent endoscopic resection experienced recurrence, metastasis, or death. The Japanese Gastric Cancer Treatment Guidelines (6th edition) stipulate that radical tumor removal must be performed in differentiated gastric cancer when the invasion depth is > 500 μm [3]. Thus, the treatment of GEN FGML is challenging. On the one hand, surgical resection may not be an advisable treatment for GA-FG with an invasive depth of > 500 μm [16]. In contrast, GA-FGM was more aggressive than GA-FG. Therefore, endoscopic treatment guidelines for common gastric adenocarcinomas should be followed to treat GA-FGM. Hence, future studies on GEN-FGMLs should establish standardized endoscopic treatment and follow-up protocols.

## Conclusion

In conclusion, GEN-FGML is a rare type of well-differentiated tumors possibly originating from dedifferentiated chief cells, with typical histopathological and molecular features. GEN-FGMLs are a group of well-differentiated gastric tumors with favorable biological behaviors, low cellular atypia, and low proliferation. It can be divided into OGA, GA-FG, and GA-FGM subtypes. Immunohistochemistry is critical for confirming diagnosis. The Ki-67 proliferation indices and lesion size may help differentiate between OGA, GA-FG, and GA-FGM, especially in biopsy specimens. Close cooperation between pathologists and endoscopists is required to avoid misdiagnosis and overtreatment. Endoscopic resection is currently the mainstream initial treatment for GEN-FGMLs.

## Data Availability

The raw data supporting the conclusion of this article will be made available by the authors, without undue reservation.
