# Supplementary material for: Gastric epithelial neoplasm of fundic-gland mucosa lineage: representative of the low atypia differentiated gastric tumor and Ki67 may help in their identification
Source: Pathol Oncol Res. 2024 May 30;30:1611734. doi: 10.3389/pore.2024.1611734 (PMC11169639; doi:10.3389/pore.2024.1611734)
Supplement: Supplementary file 1 [file Table1.docx]

| Table S1：Antibodies for immunohistochemical analysis | | | | | | |
| --- | --- | --- | --- | --- | --- | --- |
| Markers | Company | Clone | Dilution | Antigen retrival | Incubation condition(min,℃) | Assessment |
| MUC2 | Maixin Biotechnology Co. LTD | M53 | Pre-diluted | EDTA PH8 | 30,32 | C |
| MUC5AC | Maixin Biotechnology Co. LTD | 45M1 | Pre-diluted | EDTA PH8 | 30,32 | C |
| MUC6 | Maixin Biotechnology Co. LTD | MX110 | Pre-diluted | EDTA PH8 | 30,32 | C |
| CD10 | Maixin Biotechnology Co. LTD | MX002 | Pre-diluted | EDTA PH8 | 30,32 | M,C |
| CgA | Maixin Biotechnology Co. LTD | MX018 | Pre-diluted | EDTA PH8 | 30,32 | C |
| Syn | Maixin Biotechnology Co. LTD | MX038 | Pre-diluted | EDTA PH8 | 30,32 | C |
| CD56 | Maixin Biotechnology Co. LTD | MX039 | Pre-diluted | EDTA PH8 | 30,32 | M |
| Desmin | Maixin Biotechnology Co. LTD | MX046 | Pre-diluted | EDTA PH8 | 30,32 | C |
| p53 | Roch | DO-7 | Pre-diluted | EDTA PH8 | 32.37 | N |
| pepsinogen-I | Maixin Biotechnology Co. LTD | 7G3 | Pre-diluted | EDTA PH8 | 30,32 | C |
| H+/K+-ATPase | Maixin Biotechnology Co. LTD | C-4 | Pre-diluted | EDTA PH8 | 30,32 | C |
| Ki67 | Maixin Biotechnology Co. LTD | MX 006 | Pre-diluted | EDTA PH8 | 30,32 | N |
| N: nuclear, C: cytoplasmic, M: membraneous | |  |  |  |  |  |
